# Editorial: Mitochondrial remodeling and dynamic inter-organellar contacts in cardiovascular physiopathology-Volume II

**DOI:** 10.3389/fcell.2023.1240207

**Published:** 2023-06-22

**Authors:** Valentina Parra, Giovanni Monaco, Giampaolo Morciano, Gaetano Santulli

**Affiliations:** ^1^ Advanced Center of Chronic Diseases (ACCDiS), Facultad de Ciencias Químicas y Farmacéuticas, Universidad de Chile, Santiago, Chile; ^2^ Departamento de Bioquímica y Biología Molecular, Facultad de Ciencias Químicas y Farmacéuticas, Universidad de Chile, Santiago, Chile; ^3^ Center for Innovation and Stimulation of Drug Discovery (CISTIM), Leuven, Belgium; ^4^ Section of Experimental Medicine, Laboratory for Technologies of Advanced Therapies (LTTA), Department of Medical Sciences, University of Ferrara, Ferrara, Italy; ^5^ Maria Cecilia Hospital, GVM Care and Research, Cotignola, Italy; ^6^ Division of Cardiology, Department of Medicine, Albert Einstein College of Medicine, Wilf Family Cardiovascular Research Institute and Einstein Institute for Aging Research, New York, NY, United States; ^7^ Department of Molecular Pharmacology, Einstein-Sinai Diabetes Research Center (ES-DRC), Fleischer Institute for Diabetes and Metabolism (FIDAM), Montefiore University Hospital, New York, NY, United States; ^8^ International Translational Research and Medical Education Academic Research Unit (ITME), Department of Advanced Biomedical Sciences, “Federico II” University, Naples, Italy

**Keywords:** mitochondria, intracellular signaling, cell death, cardiovascular disease, calcium

Inter-cellular and inter-organellar communications are required to maintain homeostasis in complex organisms (Jain and Zoncu, 2022). Most of subcellular organelles evolved as entities needing connections, rather than separate units with unitary functions. Indeed, besides the anterograde and retrograde communication between the nucleus and organelles which involves gene transcription regulation, there is ample evidence that they come into contact with each other through several physical contact sites (Scorrano et al., 2019). Usually, such sites are represented by dynamic changes of membranes juxtaposition of neighboring organelles with specific purposes: the exchange of biological material and thus, information. Information that is required to coordinate a series of signaling pathways to sustain life.

Over the years, the understanding of inter-organellar communications has been thoroughly investigated and eventually transformed our view of cellular physiology (Quirós et al., 2016; Ghai et al., 2017; Lopez-Crisosto et al., 2017; Shai et al., 2018). Although the vesicular transport (import and export) has been described as the main system for material exchange (Bonifacino and Glick, 2004; Di Mambro et al., 2023), not all biomolecules follow this route (Lev, 2012). Non-vesicular lipid trafficking (Kaplan and Simoni, 1985; Urbani and Simoni, 1990; Heino et al., 2000) and spatio-temporal calcium (Ca^2+^) transfer (Giorgi et al., 2018) are just a couple of notable examples. These communication systems are mutually coordinated and actively participate to the intracellular signaling and the transcriptional program.

In the cardiovascular setting, mitochondrial signaling acquires considerable importance for countless reasons that can be roughly summarized with the functional role played for ATP production, in the so-called ATP cycling in cardiac contraction, as well as in Ca^2+^ handling (Gambardella et al., 2018). This second messenger is able to induce cell death and tissue function loss as consequence of the mitochondrial permeability transition pore (PTP) opening (Campo et al., 2017; Morciano et al., 2021a) and the activation of Ca^2+^-dependent proteases inducing hypercontracture (Neuhof and Neuhof, 2014). Given the critical role of these pathways for cell survival, mitochondrial dysfunctions have been disclosed as key hallmarks of cardiovascular diseases (CVDs). Also, being ATP generators and biosynthetic centers, mitochondria are important for cell metabolism with pathophysiological phenotypic features highly dependent on their morphology (Wai and Langer, 2016; Morciano et al., 2022).

In this Research Topic, Mostafavi et al. investigated the degree of mitochondrial remodeling accompanying the metabolic switch occurring in cardiomyocyte differentiation. In this regard, mitochondria from human pluripotent stem cells (hPSCs) and terminally differentiated cardiomyocytes have been evaluated by assessing differences in their number, morphology, membrane potential and the activity of the respiratory chain. Differentiated cells displayed a greater efficiency in energy production (ATP) and a greater dependence on oxidative phosphorylation (OXPHOS) with a more electronegative membrane potential despite a decrease in the number, DNA levels and mitochondrial biogenesis. These investigations, in concert with examining the impact of mitochondrial remodeling on inter-organellar dynamics, are fundamental in order to gain pathologically relevant insights.

Vásquez-Trincado et al. provided further evidence on the pivotal role played by mitochondria and mitochondrial signaling in metabolic disorders linked to CVDs. Indeed, they demonstrated how lipotoxic stress, induced by fatty acid overload, triggered cardiac hypertrophy and insulin desensitization by increasing E3 ubiquitin ligase MUL1 expression, a protein localized at mitochondria. Lipotoxic stress-induced MUL1 activation impaired some mitochondrial features by promoting organelle fragmentation through dynamin-related protein 1 (DRP1) and by decreasing mitofusin 2 (MFN2) expression. MUL1 would be required for this process as its silencing prevents cardiac hypertrophy and metabolic impairment. Further investigations on this process can lead to the identification of a reliable therapeutic target for metabolic diseases.

In the field of organelles communication, mitochondria interact at multiple levels to communicate with other organelles. Noteworthy examples are described by the existence of mitochondria—nucleus contact sites (Desai et al., 2020), mitochondria—associated membranes (MAMs) (Morciano et al., 2018) and mitochondria—plasmalemma contacts (Montes de Oca Balderas, 2021). Additionally, Guajardo-Correa et al. summarized the importance of mitochondria in CVDs by highlighting a key aspect of their connection with the nucleus. In detail, Estrogens modulation of cardiovascular physiology has been revealed as one of the most potent cardioprotective factor in humans. Multiple evidence found estrogen signaling pathways to involve mitochondria, mainly in antioxidant defense mechanisms, by reducing mitochondrial reactive oxygen species (ROS) and increasing mitochondrial antioxidant enzymes (Lynch et al., 2020). Estrogens are also potentially able to improve cellular Ca^2+^ handling, which is essential for heart contraction and relaxation (Jiao et al., 2020). How these pathways are inter-linked and intracellularly signaling to the nucleus is of growing interest. Although estrogens have pleiotropic effects, most of them are described in the nuclear—mitochondrial anterograde and retrograde communication, such as the estrogen–mediated transcriptional activity of many master regulators of mitochondrial pathways. Among them, PGC-1α, that in turn may affect fatty acid oxidation (FAO), tricarboxylic acid (TCA) cycle, and OXPHOS. PGC-1α related pathways result to be impaired in CVDs and can be finely regulated through estrogen receptors (ERs) localized on the membranes of several organelles. On the other hand, retrograde signaling also exists, and is mainly mediated by mitochondrial energetic deprivation (Hu et al., 2011), ROS stress responses (Tan et al., 2008), Ca^2+^-dependent signaling (Novotny et al., 2009), and by mitochondrial unfolded protein response (mtUPR) (Germain, 2016).

Finally, the review authored by Pedriali et al. expanded the current view on mitochondrial connections moving the focus on other points of contacts, especially those established with either the SR or the sarcolemma. The architecture of MAMs in physiology is dynamic and aims to optimize Ca^2+^ transfer (and not only) from SR to mitochondria to support cell bioenergetics. This aspect is so important for cell survival that an impairment in the architecture of MAMs is at the basis of many molecular pathways in cardiac diseases (Dridi et al., 2022; Morgado-Cáceres et al., 2022). A striking example occurs in ischemia/reperfusion (I/R) injury and heart failure, in which many studies supported the hypothesis that mitochondria are subjected to an overload of Ca^2+^, an event triggering cell death through the PTP opening (Morciano et al., 2021b). At the basis of these phenomena, an impaired interaction between SR and mitochondria occurs; here, a pharmacological or genetic approach in reducing SR-mitochondria tethering would correlate with a decrease in Ca^2+^ transfer and thus a significant lower amount of myocardial cell death. Also, a close proximity between mitochondria and cardiomyocytes gap junctions have been described (Forbes and Sperelakis, 1982) with a prominent role in I/R (Rodriguez-Sinovas et al., 2004; Fridolfsson et al., 2012).

In conclusion, mitochondrial signaling, especially the one established by nuclear-, SR- and sarcolemma-connections is a growing field of research (Kerkhofs et al., 2019) and may actually represent a fertile field for promising therapeutic purposes.
